# Bronchopulmonary dysplasia - an overview about pathophysiologic concepts

**DOI:** 10.1186/s40348-015-0013-7

**Published:** 2015-02-26

**Authors:** Sophie Niedermaier, Anne Hilgendorff

**Affiliations:** Comprehensive Pneumology Center (CPC), Helmholtz Zentrum München, Member of the German Center for Lung Research (DZL), Munich Max-Lebsche-Platz 31, 81377 Munich, Germany; Dr. von Hauner Children’s Hospital, Ludwig-Maximilians University Munich, Munich, Germany

**Keywords:** Bronchopulmonary dysplasia, Neonatal chronic lung disease, Inflammation, Mechanical ventilation, Oxygen, Extracellular matrix, Lung development, Alveolarization, Vascularization

## Abstract

Neonatal chronic lung disease in the preterm infant, i.e. bronchopulmonary dysplasia (BPD) is characterized by impaired pulmonary development with its effects persisting into adulthood. Triggered in the immature lung by infectious complications, oxygen toxicity and the impact of mechanical ventilation, a sustained inflammatory response, extensive remodeling of the extracellular matrix, increased apoptosis as well as altered growth factor signaling characterize the disease. The current review focuses on selected pathophysiologic processes and their interplay in disease development. Furthermore, the potential of both, acute and long-term changes to the pulmonary scaffold and the cellular interface in concert with dysregulated growth factor signaling to affect aging and repair processes in the adult lung is discussed.

## Introduction

With the increasing number of extremely premature preterm infants discharged from neonatal intensive care over the last decades, the development of neonatal chronic lung disease (nCLD), also known as bronchopulmonary dysplasia (BPD), accounts for a significant number of all pulmonary diseases in early infancy. Although major advances were made with respect to perinatal care, including surfactant replacement therapy, induction of lung maturation by antenatal corticosteroids, and improved invasive and non-invasive ventilation strategies, the incidence of BPD has not changed over the last decade [1]. Due to differences in patient population and infant management practices, numbers vary between newborn care centers and reach up to 68% in the group of infants between 22 and 28 weeks of gestation [2]. For Europe, the MOSAIC study group found 10% to 20% of all infants born between 23 and 31 weeks postmenstrual age (PMA) to develop BPD [3].

The disease results in adverse long-term pulmonary outcome that may persist into adulthood and is associated with an increased risk for coexistent impairments in neurocognitive development underlining the importance of BPD on morbidity in this patient population [4].

Clinically, BPD is defined by the need for supplemental oxygen and/or ventilator support for greater than 28 days (mild) or beyond 36 weeks PMA (moderate and severe) [5]. Symptoms are primarily due to alveolar hypoventilation and an impaired respiratory gas exchange, with hypoxemia and hypercapnia resulting in increased work of breathing and clinical signs of dyspnea. The mismatch of ventilation and perfusion resulting from a reduction in alveolar surface area as well as impaired vessel growth and function often precedes the development of sustained impairments including pulmonary hypertension, a common complication of the disease [5]. Furthermore, survivors of BPD frequently develop long-term respiratory morbidity, including increased airway hyperreactivity and decreased lung function, as well as a compromised pulmonary immune response, resulting in a greater risk for hospital re-admission due to respiratory tract infections in the first years of life [6].

Histopathological data from human tissue were mainly obtained from the lungs of late preterm infants in the pre-surfactant era, where the so called ‘old’ BPD was characterized by fibrotic changes and the side-by-side of over-inflation and atelectasis occurring after long-term ventilation and exposure to high oxygen concentrations. Although some of the characteristics are still seen in patients that now benefit from prenatal steroid treatment and surfactant therapy [7], the ‘new’ BPD is mainly characterized by the combination of alveolar hypoplasia and disrupted vascular development as well as saccular wall fibrosis, with minimal airway injury [8]. The histopathological picture is associated with sustained inflammatory changes, extensive remodeling of the extracellular matrix, and imbalanced growth factor signaling [9].

Clinical studies, supported by findings from experimental models, have identified and confirmed both pre- and postnatal risk factors associated with the characteristic impairment in lung development observed in infants with BPD. Key pathophysiologic mechanisms are triggered by infections occurring both *in utero* and *ex utero* [10], oxygen toxicity [11] and the impact of mechanical ventilation (MV) [12]. These risk factors act on a functional and structural immature lung beyond the individual genetic background, with impaired pre- and postnatal somatic growth in concert with hormonal dysregulation and male gender further adding to the risk for BPD development [3,6,13].

**Figure 1:**
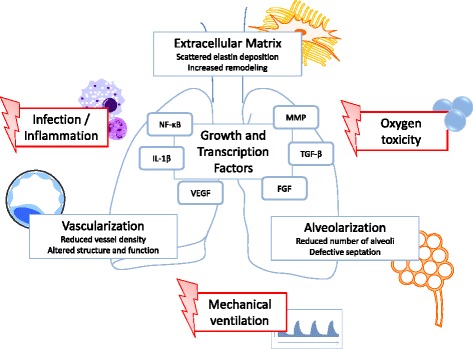
**Orchestration of risk factors and pathophysiologic variables with anticipated treatment potential in the development of BPD.**

This review will focus on key pathophysiologic mechanisms leading to BPD, their interaction with one another, and the potential contribution to long-term lung pathology. Figure 1 depicts a schematic overview of the reviewed processes critical for disease development.

## Review

### Pathomechanisms

Intra-amniotic, congenital and nosocomial infections, oxygen toxicity, and MV contribute to the onset and perpetuation of an inflammatory response in the premature lung, characterizing the development of BPD [10-12]. This inflammatory process is indicated by the influx of neutrophils into the lung followed by macrophage recruitment [10,14] as measured in the bronchoalveolar lavage (BAL) fluid obtained from premature infants developing BPD. The characteristic cytokine profile in the lungs of infants developing BPD includes elevated levels of interleukin (IL-) 8, IL-1β, IL-6, tumour necrosis factor (TNF)-α, monocyte chemo-attractant proteins, MCP-1, MCP-2 and MCP-3, as well as the macrophage inflammatory proteins, MIP-1a and MIP-1b and decreased expression of IL-10, pointing towards a disturbed balance of pro- and anti-inflammatory factors [10]. The recruitment and trans-endothelial migration of inflammatory cells is associated with the release of enzymes with tissue-damaging effects, cellular apoptosis, and the dysregulation of central signaling pathways in the lung as indicated by an increase in transforming growth factor (TGF)-β1 and the dysregulation of transcription factors such as nuclear factor (NF)-kB [10]. TGF-β activation, observed induced by prenatal infections, oxygen exposure as well as long- or even short term ventilation as observed in newborn mice, induces apoptosis and alters both cell proliferation and migration [15]. While activation of NF-kB in the adult mouse is known to exhibit pro-inflammatory effects often resulting in apoptosis, its role in the immature lung extends to anti-inflammatory as well as pro-developmental properties [14,16]. The perturbation of central signaling pathways by the described inflammatory response is linked to the disruption of airway-branching and impaired development of epithelial, mesenchymal and endothelial cell structures, culminating in a failure of lung development as shown in fetal mice [14,15].

Both, the presence of infections as well as the impact of shear stress and oxygen toxicity, independently increase lung elastase and protease activity in the developing lung, due to the accentuated release from cellular and matrix sources. Interestingly, in the newborn rat, the impact of MV and hyperoxia leads to both common and distinct extracellular matrix (ECM) changes with respect to collagen deposition, increase in interstitial thickness, and elastin content [11]. The (fatal) combination of ECM remodeling together with the increase in apoptosis and the decrease in lung cell proliferation [11] induced by alterations in growth factor signaling [17], impairs pulmonary epithelial, endothelial and mesenchymal cell survival and differentiation, leading to disrupted alveolar and capillary development.

#### Extracellular matrix

The lung extracellular matrix plays an essential role in lung development since it functions as the scaffold for developing alveoli and vessels [18]. Abnormalities in lung ECM turnover and impaired structural organization significantly contribute to the development of BPD as shown by clinical and experimental studies. Scattered elastin deposition, increased elastin breakdown and defective septation are observed in infants with BPD and in animal models of the disease, e.g. by the use of MV in newborn mice [15]. The pathologic induction of ECM breakdown is associated with increased tropoelastin production and the scattered deposition of misassembled elastic fibres in the lung [18]. Serine proteases (e.g. trypsin), neutrophil proteases, matrix metalloproteinases (MMP) and the papain family of proteases (e.g. cathepsin B, H, K, L and S) initiate and further regulate the remodeling process of the ECM. While expression of proteases such as MMP-2 and -9 are needed to enable cell migration and stimulate physiologic remodeling of the ECM in the developing lung on the one hand [18], excess secretion initiates disease pathology through accentuated ECM breakdown resulting in the destruction of the intact alveolo-capillary interface on the other [10]. In line with this, imbalanced expression of different MMP subtypes is associated with BPD development [10,18] and hyperoxia has been shown to increase pulmonary MMP-9 activity in neonatal rat lungs [11], with its expression heralding the chronic and regenerative phase of BPD [18].

The indicated imbalance of pulmonary proteases and their inhibitors in the immature lung can be attributed to their enhanced release from the matrix, as well as an increased production by resident cells such as smooth muscle cells and inflammatory cells recruited to the injured lung. In addition, the immaturity of the lung results in the insufficient expression of anti-proteases. The resulting perpetuation of this remodeling process leads to impaired vessel development and alveolarization as well as the reduction in functional abilities of different cell types reflected by, i.e. decreased surfactant production and turn-over [11]. Similarly, the increased presence of toxic oxygen metabolites further stimulates the release of proteases such as elastase, myeloperoxidase and xanthine oxidase from the ECM as well as their activation [10,18]. These processes are further enhanced by the relative deficiency of anti-oxidants such as superoxide dismutase or glutathione peroxidase rendering the immature lung to an increased vulnerability to oxidative stress [10]. Pathologic ECM remodeling and the subsequent disruption of the intact pulmonary scaffold development not only hinders the formation of the alveolar and vascular network but exceeds the effect of mere structural changes by impacting cell fate [18].

Closely connected to the changes in the ECM, the TGF-β superfamily, including the bone morphogenic proteins (BMP), plays an important role in lung development by influencing the cellular composition of the lung via the regulation of endothelial and epithelial cell survival as well as influencing ECM production and remodeling [19]. TGF-β signaling, required for physiologic lung development, is activated in the vascular and airway smooth muscle and the alveolar and airway epithelium throughout late lung development [20]. Primarily secreted by monocytes and macrophages recruited to the lung, TGF-β can also be released from the ECM where latent TGF-β binding proteins link TGF-β to the fibrillins. An imbalance of signaling molecules of the TGF-β/BMP signaling pathways has been described in various animal models of BPD induced by the exposure to MV and/or hyperoxia. In these models, excess TGF-β activation leads to apoptosis and the imbalance of proliferation and migration in various cell types including endothelial cells and (myo)fibroblasts alveolar and vascular hypoplasia characterizing BPD [21].

#### Alveolarization

From the pseudoglandular to the canalicular stage of lung development, the appearance of primary and secondary crests marks an essential step in lung growth. Here, the epithelial-mesenchymal crosstalk is known to be critically involved in the gradual process. The resulting formation of new alveoli establishes the appropriate interface with the developing capillary bed allowing for sufficient gas-exchange [20]. In the contrary, lung architecture in BPD is characterized by alveolar simplification with a reduced number of enlarged alveoli [5], caused by apoptosis of cells critical for alveolar and vessel formation as well as altered cell fate and function in the face of extensive ECM remodeling.

The (myo-) fibroblast, signaling through the platelet derived growth factor (PDGF), is a critical driver of secondary septation including its contribution to elastin production. Early abrogation of PDGF signaling therefore results in a failure of alveolarization indicated by an emphysematous lung phenotype [18].

Other growth factors orchestrating the cross-talk between the epithelial and mesenchymal cell compartment are members of the fibroblast growth factor family (FGF), essential mediators of alveolar morphogenesis and stimulators of elastin synthesis [20]. Expressed by mesenchymal cells, FGF-10 promotes the stereotyped sequence of lung bud outgrowth and epithelial branching and proliferation through tightly regulated signaling mechanisms [22]. FGF-7 stimulates proliferation of epithelial cells and appears to play a role in the prevention of lung epithelial damage due to external injury [18]. The disrupted process of secondary septation as well as the malassembly of the elastic fiber induced by MV and hyperoxia can in part be explained by the perturbation of the FGF signaling pathway through the activation of different inflammatory response variables, i.e. NF-kB, IL-1β, TNF-a or toll-like receptors (TLR)-2 and TLR-4 [18,22]. These inflammatory markers have been shown to reduce FGF 10 expression *in vitro* which might contribute to reduced and defective alveolar branching [22].

On the transcriptional level, epithelial cell survival and differentiation is developmentally regulated by a multitude of different factors including FOX proteins and GATA6 [20]. In a baboon model of BPD using premature delivery and oxygen supplementation to induce disease pathology, the hypoxia-inducible factor (HIF) 1α, but not HIF 2, was found to be downregulated in the pulmonary tissue. Underlining its potential role in BPD pathophysiology, stimulation of HIF by inhibition of HIF degrading proteins in the same model promoted alveolarization and angiogenesis thus leading to improved lung function [23]. With respect to its cell specific effects, HIF, in addition to activating downstream target genes that increase oxygen delivery, acts on the vascular endothelial growth factor (VEGF), well known for its role in mediating angiogenesis, but furthermore exhibiting critical impact on epithelial cell survival and differentiation, thereby influencing surfactant production [17]. Thus, intrauterine VEGF-treatment of HIF-2α-deficient newborn mice that developed severe respiratory failure immediately after birth, improved impaired gas exchange and respiratory outcome by stimulating alveolar pneumocytes [24].

#### Angiogenesis

The vascular hypothesis establishes angiogenesis as a main driver of alveolarization and therefore a critical process for lung development [17]. The pulmonary capillary network grows along the buds of newly developing airways and developing canalicular and saccular airspace structures. As the airspaces grow larger in size, the capillary network expands through both sprouting and intussuscepted growth [17]. An impairment of angiogenesis indicated by fewer small vessels and their abnormal distribution, is mirrored by a disturbed alveolar structure [5]. The role of vascular development for alveolarization was further established in different animal studies, where, the inhibition of angiogenesis by the use of specific antibodies or in knock-out mice showed disabled VEGF expression or its down-stream signaling to result in disruption of the alveolarization process itself [17]. In keeping with these studies, the induction of angiogenesis protects or enhances alveolarization in animal models of BPD [21]. Reduced pulmonary expression of endothelial markers in the human lung after short term MV in contrast to reports of elevated endothelial markers after long term ventilation may indicate compensatory mechanisms or be the effect of fluctuating oxygen saturation levels in the developing lung undergoing MV and oxygen treatment [17].

The VEGF pathway is of particular importance in promoting angiogenesis in the developing lung. This cell-specific endothelial growth factor is a critical agent for vascular development enabling formation and remodeling of pulmonary vessels and alveolar structures. Its expression peaks during the early stages of lung development and decreases to adult levels in the last stage of alveolarization [17]. VEGF stimulates neovascularization at the leading edge of the branching airways, thereby connecting the development of blood vessels and airways, i.e. through the modulation of FGF signaling [22]. Over-expression of VEGF in the embryonic phase increases growth of pulmonary blood vessels but in the same time disrupts the morphogenesis of airway branches and alveolar type-I cell differentiation [17]. Decreased VEGF levels have been found in preterm infants who subsequently developed BPD [17], associated with reduced pulmonary capillary volume and impaired alveolarization in a mouse model of the disease [24]. In different animal models, VEGF expression decreased upon injury induced by hyperoxia, prolonged mechanical ventilation or endotoxin exposure [21]. Indicating a tight regulation during development, induced pulmonary VEGF levels were reported depending on the degree of hyperoxia and some studies interpreted elevated VEGF levels as an indicator of recovery from injury [11]. Destruction of alveolar septi in the adult lung after inhibition of VEGF signaling underlines its importance as a pro-survival factor [17]. Linking these changes back to ECM remodeling, BMP family members have been shown to stimulate the process of angiogenesis in the lung by increasing pro-angiogenic signaling through increasing VEGF receptor expression (VEGF-R2) [25], influencing pulmonary angiogenesis via the wingless (Wnt) pathway at the same time [26]. Abrogation of BMP signaling therefore likely leads to compromised signaling in the VEGF pathway causing and contributing to impaired lung development. Table 1 summarizes the main findings derived from animal models cited in this review.

**Table 1:** **Selection of animal models mimicking BPD**

**Study**	**Species**	**Mode of injury**	**Main finding**
Alvira et al. [27]	Mouse (PN 5, 16 weeks)	Intraperitoneal injection of lipopolysaccharides (LPS)	Persistent NF-kB activation in the fetal lung leads to reduced inflammation and apoptosis 24 h after LPS exposure in contrast to adult animals
Asikainen et al. [23]	Baboons (E 125)	MV at room air or with oxygen supplementation as calculated by oxygenation index	Enhancement of angiogenesis by activation of HIF improves lung growth and function
Blackwell et al. [14]	Mouse (E 15)	LPS in isolated macrophages	Macrophage depletion or targeted inactivation of the NF-κB signaling pathway protects airway branching from adverse LPS effects
Bland et al. [15]	Mouse (PN 5 to 7)	MV at room air or with 40% oxygen	MV results in increased elastase activity, reduced abundance of proteins regulating elastic fiber assembly and scattered deposition of elastic fibres in the lung
Compernolle et al. [24]	Mouse (E 18.5)	Transgenic mouse model (knock out of HIF 2α)	Intrauterine delivery or postnatal intratracheal instillation of VEGF stimulates surfactant synthesis and reduces respiratory distress
Iosef et al. [16]	Mouse (PN 6)	Intraperitoneal injection of blocker of NF-κB (BAY 11-7082)	NF-κB promotes physiological angiogenesis and alveolarization in the developing lung
Wallace et al. [12]	Lambs (E 125 and 132)	MV at room air	Increase in pulmonary pro-inflammatory cytokines transcription

With the limited access to human tissue samples, the pathophysiologic understanding in BPD mainly relies on the findings generated by the use of these and other experimental models in lambs, baboons, rats and mice trying to mimic characteristic histopathological findings while still mirroring important clinical conditions [7]. In the mouse model, the induction of BPD by the impact of moderate hyperoxia, i.e. 40% o2 and/or MV led to air space enlargement with widened, hypercellular septae and reduced number of alveoli and vessels [28]. Studies in rats showed focal fibrotic sites next to perivascular, interstitial, and alveolar edema upon MV [29]. Findings from a baboon model of the disease using MV in animals of different GA to induce BPD pathology fostered the ability to study different stages of the disease as well as treatment settings with potential clinical relevance. Whereas injury in more mature animals led to the development of enlarged alveoli, increased edema and abnormal abundance of elastin with only minor fibrotic changes, more immature animals developed dysmorphic microvasculature and varying degrees of fibroproliferation [30,31].

Nonetheless, although the histopathological findings in animal models resemble the changes seen in human lungs upon disease development, it has not been possible to fully imitate the pathological picture of BPD.

### Long-term outcome

Clinically, patients with BPD often suffer from respiratory exacerbations due to viral infections and asthma-like symptoms such as airway hyper-responsiveness and impaired exercise intolerance [9]. In line with this, infants with BPD require more frequent hospital readmissions compared to preterms without BPD. Radiological findings even in asymptomatic BPD survivors show changes in the peripheral airways, with interstitial fibrotic changes still present in the lungs of adult survivors of BPD [6].

Increasing evidence suggests that early pulmonary injury leading to the pathophysiologic changes characterizing BPD may contribute to premature aging of the lung [21]. The sustained and potentially irreversible alterations of structure and function affecting both ECM as well as epithelial, endothelial and mesenchymal cells likely result in impaired pulmonary function including the development of cardiovascular diseases, i.e. pulmonary hypertension, often complicating the clinical course of patients with BPD [6]. The scaffold provided by the ECM seems to hold a ‘memory function’, as studies using decellularized lungs showed cell fate to be significantly influenced by matrix composition, thereby indicating long-term implications of ECM remodeling with respect to pulmonary repair capacity and aging related processes [6,32]. With respect to the pathophysiologic changes observed in the pulmonary circulation, studies in adult mice that outgrew oxygen supplementation in the neonatal period suggest sustained changes in the pulmonary vascular structure [11,21]. Furthermore, evidence of increased oxidative stress was detected even in adolescent patients with BPD, indicating that damage to the developing lung may be associated with sustained perturbations in the pulmonary oxidative stress response [4]. The underlying differences in the equilibrium of signaling pathways in the developing lung initiating and prolonging pathophysiologic changes in patients with BPD may persist during lung maturation and need to be considered when treatment regimen are applied [27]. First reports suggest that the inhibition of gene expression in the fetal mouse lung via the NF-κB signaling pathway is associated with impaired alveolarization and may even result in epigenetic changes [14].

## Conclusions

BPD is characterized by inflammation, apoptosis of various cell types and extensive ECM remodeling. Three main risk factors, i.e. pre- and postnatal infections, hyperoxia and MV lead to the complex interaction of pro- and anti-inflammatory proteins and the extensive alterations of signaling pathways associated with growth factor imbalance. Several *in vitro* and *in vivo* studies have contributed to our understanding of molecular pathways involved in disease development and their critical interplay, leading to the identification of potential treatment targets. Most importantly, the use of experimental approaches and their (partial) verification in clinical studies have connected critical pathways involved in the processes of angiogenesis, alveolarization and ECM remodeling in the context of BPD development [21]. Although the indicated pathways impact early postnatal lung development on different levels, it has furthermore been demonstrated that the effects are not only acute and time-restricted. In the contrary, permanent changes in gene regulation and significant structural changes, are causing long-term changes in pulmonary function [4]. Further studies are needed to study the effect of promising treatment strategies with respect to their potential to influence long-term outcome. These studies have to take the limitations of the experimental models into account that are widely used to generate our pathophysiologic understanding.
